# Improving Cure Rates for Patients with Newly Diagnosed Large B-Cell Lymphomas: Targeted Therapies for High-Risk Pathologic Subgroups as Defined by Clinical Laboratory Testing

**DOI:** 10.3390/cancers17010018

**Published:** 2024-12-24

**Authors:** Daniel J. Landsburg

**Affiliations:** Abramson Cancer Center, Philadelphia, PA 19104, USA; daniel.landsburg@pennmedicine.upenn.edu

**Keywords:** diffuse large B-cell lymphoma, targeted therapy, immunohistochemistry, fluorescence in situ hybridization, next-generation sequencing

## Abstract

Background/Objectives: Diffuse large B-cell lymphoma (DLBCL) and high-grade B cell lymphoma (HGBL) comprise the majority of large B-cell lymphomas (LBCL), and approximately two-thirds of patients diagnosed with these LBCLs are cured following treatment with first-line immunochemotherapy. While the International Prognostic Index (IPI) score is a validated prognostic tool used for patients treated with rituximab, cyclophosphamide, doxorubicin, vincristine, and prednisone (R-CHOP), there is a growing body of evidence that suggests that LBCL tumor features, which can be detected by clinical laboratory testing, can predict patient survival following first-line immunochemotherapy. Conclusions: Clinical laboratory testing may also allow for rational identification of targeted agents that can be added to first-line immunochemotherapy for high-risk, pathologically defined subsets of LBCL patients, and this approach may result in better survival outcomes for the entire LBCL patient population as compared with adding pathologically “agnostic” agents for those defined as high risk by IPI score.

## Simple Summary

While many patients diagnosed with large B cell lymphomas (LBCL), specifically diffuse large B cell lymphoma (DLBCL) will be cured following treatment with first-line immunochemotherapy, those who are not are likely to die from complications of this disease. This discrepancy in outcome suggests biologic heterogeneity of this common lymphoid malignancy, and LBCL tumors can be both classified and risk-stratified by testing available in the clinical laboratory. Such testing may also identify opportunities to add targeted agents to first-line immunochemotherapy in homes of improving survival for patients whose tumors harbor high-risk pathologic features, examples of which are offered. Whether more complex comprehensive genomic analyses can be applied in clinical practice and enhance this effort remains to be determined.

## 1. Introduction

Diffuse large B-cell lymphoma (DLBCL) is the most common subtype of non-Hodgkin lymphoma, representing approximately one-third of all cases, with an incidence of 7.2 cases per 100,000 in the United States. In terms of demographic information, DLBCL is associated with female sex, non-Hispanic white race/ethnicity, and older age. Risk factors for the development of DLBCL include immunodeficiency syndromes, autoimmune disease, environmental exposures, and rarely genetic syndromes [1].

Large B-cell lymphomas, including DLBCL and high-grade B-cell lymphoma (HGBL), can be diagnosed through pathologic assays available in clinical laboratories. These include immunohistochemical staining (IHC), fluorescence in situ hybridization (FISH), and next-generation sequencing (NGS). IHC has been primarily useful in determining tumor cell of origin (COO), which designates the compartment within the lymph node in which lymphomagenesis occurs and can distinguish germinal center B (GCB) from non-GCB as per the Hans algorithm [2] and others with approximately 90% concordance between these IHC-based algorithms and gene expression profiling (GEP) [3], which is considered the gold standard for COO designation and not prone to issues such as qualitative assessment of expression. Patients diagnosed with non-GCB or activated B-cell (ABC) DLBCL are reported to experience an inferior prognosis when treated with rituximab, cyclophosphamide, doxorubicin, vincristine, and prednisone (R-CHOP) as compared with those with GCB DLBCL [4,5], which has led to the design of numerous clinical trials evaluating the benefit of the addition of novel agents to R-CHOP in patients with newly diagnosed non-GCB/ABC DLBCL, including bortezomib [6,7], ibrutinib [8], and lenalidomide [9,10], although the randomized trials revealed no significant survival advantage as compared with R-CHOP alone. More recently, IHC has been used to identify cases of double expressor lymphoma (DEL), defined as LBCL cases without rearrangement of *MYC* and *BCL2* with increased expression of MYC and BCL2 protein, with typical cutoffs of 40% for MYC and 50% for BCL2 based upon large retrospective series [11,12]. Evaluation for COO and DEL status by IHC are standard components of the LBCL tumor pathologic evaluation, although at least National Comprehensive Cancer Network (NCCN) guidelines do not support first-line treatment selection based upon these features [13].

FISH is most commonly utilized to assess for the presence of double-hit lymphoma (DHL) harboring *MYC* and *BCL2* rearrangements, whether by simultaneous testing for both rearrangements or reflex testing for *BCL2* rearrangement if *MYC* rearrangement is detected first. For this entity, which is now defined as DLBCL/HGBL with *MYC* and *BCL2* rearrangements in the World Health Organization (WHO) 5th edition [14], consideration of intensive first-line immunochemotherapy regimens, such as dose-adjusted etoposide, prednisone, vincristine, cyclophosphamide, doxorubicin, and rituximab (DA-EPOCH-R), is recommended in NCCN guidelines [13], although this is supported largely by retrospective data that suggest a benefit to these regimens over R-CHOP [15,16]. Of note, a randomized trial of R-CHOP vs. DA-EPOCH-R for patients with newly diagnosed stage II-IV LBCL without a history of indolent lymphoma showed no difference in the primary endpoint of 2-year progression-free survival (PFS); however, limited numbers of patients with DEL and DHL were enrolled, and so this trial was unable to determine if the use of DA-EPOCH-R could result in a survival benefit for patients diagnosed with these subsets of LBCL [17].

Targeted NGS is less widely utilized for the evaluation of LBCL tumors in clinical practice due to more limited availability of this assay in clinical laboratories, as well as the lack of utility in the diagnosis or management of LBCL cases at this time. Additional issues include variation in assay type (amplicon-based sequencing vs. capture hybridization-based sequencing), as well as variation in genes analyzed as well as types of alterations (single nucleotide variants vs. copy number alterations) across platforms. Comprehensive genomic analysis in experimental laboratories has led to the discovery of genetic subgroups of LBCL with differential survival outcomes following treatment with R-CHOP [18,19], with subsequent development of the LymphGen algorithm, which incorporates the presence of primarily single nucleotide variants and copy number alterations to classify approximately two-thirds of LBCL tumors into genetic subgroups [20]. However, the genes analyzed in clinical laboratory panels do not typically mirror those required for the LymphGen algorithm; thus, the LymphGen algorithm is not routinely applied in clinical practice. Isolated mutations or copy number changes may be prognostic in this patient population, the most notable of which is *TP53* [21,22,23].

While we have multiple assays available in the clinical laboratory that can identify distinguishing features of LBCL tumors, at best, they have so far amounted to enhancing our ability to risk stratify tumors and identify patients who are likely to experience differential survival outcomes following treatment with standard first-line therapies, similar to that of the International Prognostic Index (IPI). But unlike the IPI score, which may not be the optimal prognostic score to utilize in patients treated with R-CHOP given the ability of the National Cancer Comprehensive Network (NCCN)-IPI to better identify a high-risk subgroup with a lower survival rate [24], these pathologic assays lend themselves to identifying targeted therapies that may improve outcomes for certain high-risk LBCL patients if added to standard first-line therapy. Investigating this approach for a few high-risk pathologic LBCL subgroups with significant incidence may result in improved survival for the entire LBCL population.

## 2. Non-GCB/ABC DEL

One example of this is the addition of Bruton tyrosine kinase inhibitors (BTKis) to immunochemotherapy in patients with newly diagnosed non-GCB/ABC DEL. The frequency of DEL in newly diagnosed DLBCL patients is approximately 20–35%, with approximately two-thirds to three-quarters of DEL tumors classified as ABC COO [11,12,25], resulting in estimates of non-GCB/ABC DEL of 15–25%. While a phase 2 study of ibrutinib in relapsed/refractory (R/R) DLBCL demonstrated a favorable response rate in patients with ABC COO tumors [26], the addition of ibrutinib to R-CHOP did not result in improved event-free survival (EFS) or overall survival (OS) as compared with R-CHOP alone for patients with newly diagnosed DLBCL enrolled in the randomized phase 3 PHOENIX study, although this overall finding was likely driven by poor tolerance of the experimental arm in patients aged ≥60 years due to adverse events including febrile neutropenia, diarrhea, neutropenia, pneumonia, and lung infection, leading to R-CHOP discontinuation in over one-third of patients [8]. However, an analysis of patients enrolled in PHOENIX categorized by the presence or absence of high expression of MYC/BCL2 by RNA sequencing revealed a statistically significant improvement in EFS for all patients with high MYC/BCL2 expression treated with ibrutinib + R-CHOP as compared with R-CHOP (estimate of approximately 70% vs. 55% at 2 years), as well as a significant improvement in EFS (estimate of approximately 75% vs. 50% at 2 years) and OS (estimate of approximately 90% vs. 70% at 2 years) for the subset of patients age <60 years [27]. This finding is supported by evidence of increased B-cell receptor signaling activity by quantitative immunofluorescence in tissue samples from patients with newly diagnosed DEL as compared with non-DEL [28], as well as a retrospective study reporting an overall response rate (ORR) of 60% and complete response rate (CRR) of 47% in R/R non-GCB DEL patients treated with ibrutinib monotherapy [29]. Interestingly, a prospective study of nearly 50 patients with DEL treated with zanubrutinib + R-CHOP reported an estimated 1-year PFS rate of 85% [30]. The result of the randomized phase 3 study of acalabrutinib + R-CHOP vs. R-CHOP alone in patients with newly diagnosed non-GCB DLBCL (ESCALDE, NCT04529772) may further address the efficacy and safety of the addition of BTKi therapy in combination with R-CHOP in patients with DEL.

## 3. *TP53* Mutation

Another example is the addition of DNA methyltransferase inhibitors (hypomethylating agents, HMA) to immunochemotherapy for patients with newly diagnosed LBCL with *TP53* mutation. *TP53* mutations have been detected in approximately 20–35% of newly diagnosed LBCL cases [21,22,23], with approximately 85–90% of mutations resulting in loss of function (LOF) of p53 protein [21,23]. Estimates of 2-year PFS for patients with TP53-mutated LBCL included in these studies approximate 50%, with inferior survival noted for the subset of patients with LOF mutations as well as those treated with intensive first-line immunochemotherapy [23]. While clinical trials specifically enrolling patients with *TP53*-mutated LBCL have not been designed, there is evidence that decitabine added to R-CHOP may improve outcomes for this patient population, as 5/5 patients with *TP53* mutations enrolled in a phase 1/2 study of decitabine + R-CHOP in newly diagnosed DLBCL patients achieved CR and all experienced PFS >2 years [31], and results from the randomized GUIDANCE-01 which randomized patients with newly diagnosed LBCL to R-CHOP vs. R-CHOP-X based upon application of a 20 gene algorithm, demonstrated an ORR of 91% in patients with *TP53*-mutated LBCL treated with decitabine + R-CHOP as compared with 60% for those treated with R-CHOP alone [32]. This finding is supported by increased serum levels of CD4+ and CD8+ T cells as well as interferon-γ in patients with *TP53*-mutated tumors treated with decitabine + R-CHOP [31], noting that *TP53*-mutated DLBCL tumors demonstrate decreased interferon signaling as well as decreased upregulation of CD8+ T cells [33]. A clinical trial of azacitidine + R-CHOP for patients with *TP53*-mutated DLCBL is enrolling in China (NCT06158399), and results of this study may be informative.

## 4. Impact of Addition of Novel Therapies to R-CHOP for the LBCL Population

**Figure 1:**
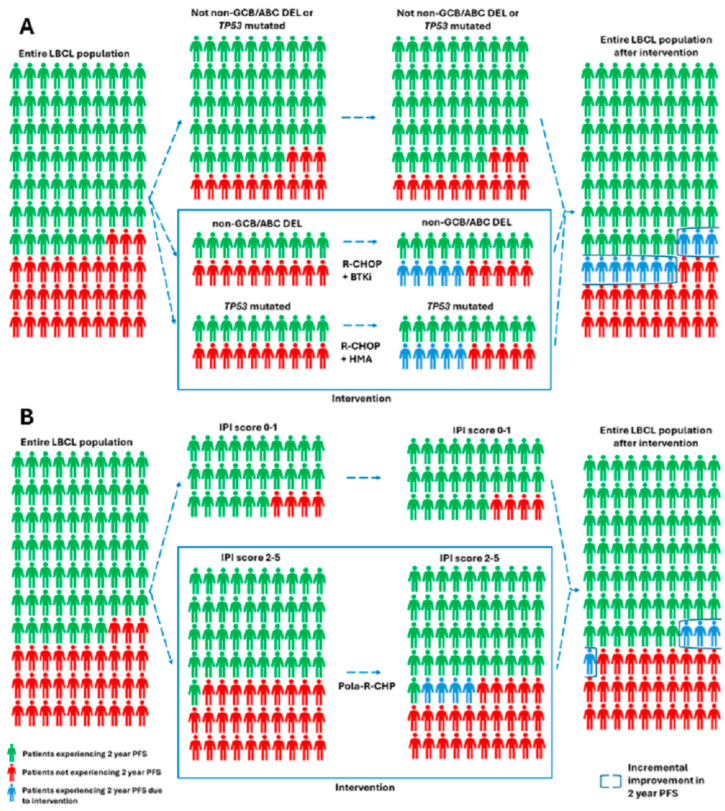
Survival outcomes for LBCL patients based on strategies for adding novel therapies to R-CHOP based upon (**A**) targeted therapies for patients of high-risk pathologic subgroups or (**B**) “agnostic” therapies for patients with high IPI scores.

If estimating that 20% of newly diagnosed LBCL patients’ tumors are non-GCB/ABC DEL and 20% *TP53*-mutated, and that adding BTKi and HMA, respectively, to R-CHOP could result in an improvement in 2-year PFS from approximately 50 to 75% for both subgroups, this would result in a 10% improvement in 2-year PFS for the entire LBCL population as depicted in panel A of Figure 1. In contrast, the 2-year PFS benefit of 6.5% for patients enrolled in the POLARIX study treated with pola-R-CHP as compared with R-CHOP [34] results in a 4% benefit for the entire LBCL patient population, if estimating that 70% of the LBCL population has an IPI score ≥2 (which was the main inclusion criteria) [35,36] as depicted in panel B of Figure 1. Interestingly, 5-year follow-up of POLARIX presented in abstract form revealed a 4% PFS benefit to pola-R-CHP over R-CHOP without evidence of overall survival benefit [37], and given that pola-R-CHP was associated with higher rates of grade 3–4 febrile neutropenia and anemia [34], it remains unclear if pola-R-CHP should be offered routinely to all patients with IPI scores ≥2.

While these projections of the benefit of targeted therapy added to R-CHOP for high-risk pathologic subgroups of LBCL may be overestimates due to the fact that there is likely some overlap between non-GCB/ABC DEL and *TP53*-mutated LBCL and not all patients may be candidates for the addition of targeted agents to R-CHOP, this approach could reasonably lead to similar if not greater improvements in PFS for the entire LBCL patient population through intervening on a smaller proportion of patients than adding a pathologically “agnostic” therapy to R-CHOP for all patients with a high-risk IPI score.

## 5. Addition of Targeted Therapies to R-CHOP Based upon Comprehensive Genomic Analysis

If the approach of adding targeted therapies to first-line immunochemotherapy for high-risk pathologic subgroups of LBCL is worth pursuing, how much more do we have to gain in identifying additional high-risk pathologic subgroups by genetic classification, and “is the juice worth the squeeze” for developing molecular assays for genetic classification that can be routinely applied in clinical practice, given the concerns of availability, turnaround time, and cost of applying more complex molecular testing to LBCL cases?

It seems likely that there is at least some overlap between some of the aforementioned high-risk pathologic subgroups of LBCL and high-risk genetic subgroups as identified by comprehensive genomic analysis. Cluster 3 and EZB genetic subtypes are both associated with poor survival outcomes in LBCL patients treated with R-CHOP [18,19], although it has been demonstrated that patients with EZB tumors only have inferior survival outcomes if also harboring DHL [20] or *TP53* LOF mutation [23]. Approximately one-third of newly diagnosed DLBCL patients enrolled in a phase 2 study of R-CHOP + tazemetostat had tumors classified as the EZB genetic subtype [38], and final results of this study will likely reveal the efficacy of this combination in this patient subgroup. Similarly, cluster 5 and MCD genetic subtypes are also both associated with poor survival outcomes in LBCL patients treated with R-CHOP [18,19], which may be improved with the addition of ibrutinib to R-CHOP [39], and there appears to be overlap between non-GCB DEL and MCD genetic subtypes, with over two-thirds of DEL cases that can be assigned a genetic subtype classified as MCD [27]. It is possible, although not yet known if the presence of DEL can risk-stratify outcomes for LBCL patients with MCD tumors treated with R-CHOP.

Furthermore, the use of genetic classifications may present challenges if attempting to add targeted therapies to R-CHOP based upon the presence of individual mutations. While *TP53* mutation is a hallmark feature of the A53 genetic classification, which has an incidence of only 6.6%, *TP53* mutations are found in approximately 20–50% of tumors characterized by other genetic classifications [20]. Therefore, the strategy of offering HMAs to patients with A53 tumors only would miss the majority of those whose tumors harbor a *TP53* mutation. Another mutation that may predict poor survival following treatment with R-CHOP is *CREBBP* [40,41,42], which is a feature of the EZB genetic classification [20]. However, the incidence of this mutation in EZB cases is only approximately 50%, such that adding a therapy targeted at *CREBBP* loss of function (such as a histone deacetylase [HDAC] inhibitor) to R-CHOP for LBCL patients with EZB tumors may be ineffective for a large proportion of these patients if, in fact, *CREBBP* mutation is a biomarker for response to HDAC inhibitors.

## 6. Conclusions/Future Directions

While it is certainly a valid effort to move forward with the development of comprehensive genetic classification assays for LBCL cases that can be applied in the clinical laboratory, which may ultimately lead to the development of treatment strategies leading to greater improvements in survival outcomes for LBCL patients than approaches discussed here, it seems as if we currently have tests at our disposal to both risk stratify and identify potential targeted therapies for patients diagnosed with high-risk pathologic subgroups of LBCL. Assays that are currently available in the clinical laboratory can help to improve survival of the entire LBCL population by the examples offered here: even treating only non-GCB/ABC DEL R-CHOP + BTKi or *TP53*-mutated LBCL with R-CHOP + HMA could result in a similar survival benefit to the entire newly diagnosed LBCL patient population as compared with adding a pathologically “agnostic” agent to R-CHOP for patients as defined by high-risk IPI score but would do so by altering treatment for 20% as compared with 70% of patients. Efforts should be made to further validate analyses performed utilizing IHC, FISH, and NGS and standardize the application of all of these assays to LBCL tumors from newly diagnosed patients, as well as develop additional clinical trials investigating targeted agents used in combination with first-line immunochemotherapy in patients with high-risk pathologic subgroups of LBCL. We should not wait for the implementation of more complex molecular assays to attempt to cure more patients with newly diagnosed LBCL when we have the tools within the clinical laboratory to do so now.
